# A Spicy Letter to the B. M. and S. Journal

**Published:** 1887-07

**Authors:** 

**Affiliations:** Per H. T. Champney; New York


### CORRESPONDENCE.

### A SPICY LETTER.

[The following is such a perfect answer to the carpers and self righteous—who see the mote in others eye, but cannot see the beam in their own, that we are induced to publish it (which we do by permission) instead of writing an editorial on the subject.—ED.]

NEW YORK, May 27, 1887.

MESSRS. CUPPLES & HURD, Boston.

*Gentlemen:*—Your printed circular referring to the admission to to the columns of the journal published by you of "laudatory notices" etc., etc., does not fit our case in the least.

We have not requested you to insert any matter referring to our preparation specifically, or in terms of approbation, so that the article we called your attention to, giving an account of a laparotomy performed by a noted surgeon and extended by him from his clinical notes, does not come within the category at all, for the case is sufficiently interesting on its own merits to command the attention of the medical fraternity, as it furnishes evidence of these capital operations in a field comparatively new, if treated with the proper antiseptic precautions and followed by an adjuvant as capable of sustaining life under such conditions as is the Fluid Food we are manufacturing. There is no more impropriety in alluding to our Food in such a connection, than in speaking of the use of Squibb's Chloroform, or Merk's Terrebene, it being a therapeutical agent simply, which in the opinion of, and in accordance with, the experience of Dr. Dickson, was entitled to be brought to the attention of his medical brethren as having materially assisted the poor patient in her terrible ordeal. We had no part in procuring the preparing of the article in question, it being voluntarily sent to us by Dr. Dickson, who desired to show his gratitude for the aid given him in the matter. We sent the matter to Dr. P. B. Porter, of Gaillard's Medical Monthly, with the request, that if compatible with his ideas of propriety as the exceedingly conservative editor of an exceedingly conservative journal, we should be pleased to see it appear in his columns. It was accepted with thanks, as a valuable contribution to the literature of laparotomy, and without reference to our being advertisers in the journal of which he was the entirely independent editor. [It was reproduced in this Journal last month.—Ed.]

If Dr. Shattuck sits up so high in the empyrean of medical dignity and is so hyper-esthetic that he cannot reproduce in his journal, a well written paper, because it gives the name of an article that proved to be of great value in a certain case, why, he is too good for this world, and should be translated to a still more exalted sphere at once. There is more rot and nonsense about the code of ethics subscribed to by some of these dainty fellows than would run a grist mill in New Hampshire. You may *talk* as you like, the *facts* are incontrovertible; you publish the B. M. & S. J. *for the money you make out of it*; Dr. Shattuck edits it for *filthy lucre*; *We make Bovinine for the same end;* our interests are thus far *identical.* Of course by lowering the tone of your journal to indiscriminate puffery of every nostrum that comes along, you would defeat the very object you are aiming at. By giving prominence, however, to articles of acknowledged merit, endorsed by leading surgeons and physicians of the best schools, you enhance the value of your own property, and instruct the thousands of readers of the *Journal* in in the use of a thoroughly legitimate food preparation.

Your advertisers sustain your publication, and without them it would not *pay a dollar*, as you well know. We have conversed quite recently with some of the most extensive advertisers in this city, and they expressed the opinion that there would be a great reaction in the matter of using the medical journals as a medium of communication with physicians, unless more palpable evidence was given of some sympathy with the objects sought to be obtained by such reputable concerns.

It is all very well to be conservative, but when that excellent quality degenerates into stupid *obstruction* in these days of celerity of thought and action, conservatism must go to the wall.

We remain, gentlemen, yours respectfully.

THE J. P. BUSH MFG. CO.
Per H. T. Champney.

